# Bilateral Psoas Haematomata Complicating Renal Transplantation

**DOI:** 10.1155/2014/678979

**Published:** 2014-01-02

**Authors:** Jacob A. Akoh, Tahawar A. Rana, Daniel Higgs

**Affiliations:** South West Transplant Centre Gastroenterology, Surgery & Renal Services Directorate, Plymouth Hospitals NHS Trust, Derriford Hospital, Plymouth PL6 8DH, UK

## Abstract

*Background*. The challenge in managing patients undergoing renal transplantation is how to achieve optimum levels of anticoagulation to avoid both clotting and postoperative bleeding. We report a rare case of severe postoperative retroperitoneal bleeding including psoas haematomata complicating renal transplantation. *Case Report*. SM, a 55-year-old female, had a past history of aortic valve replacement, cerebrovascular event, and thoracic aortic aneurysm and was on long-term warfarin that was switched to enoxaparin 60 mg daily a week prior to her living donor transplantation. Postoperatively, she was started on a heparin infusion, but this was complicated by a large retroperitoneal bleed requiring surgical evacuation on the first postoperative day. Four weeks later, she developed features compatible with acute femoral neuropathy and a CT scan revealed bilateral psoas haematomata. Following conservative management, she made steady progress and was discharged home via a community hospital 94 days after transplantation. At her last visit 18 months after transplantation, she had returned to full fitness with excellent transplant function. *Conclusion*. Patients in established renal failure who require significant anticoagulation are at increased risk of bleeding that may involve prolonged hospitalisation and more protracted recovery and patients should be carefully counselled about this.

## 1. Introduction

Measures to prevent venous thromboembolism (VTE) are well established in the management protocol of patients undergoing major surgery. Although decreased thrombogenicity is common in patients with established renal failure, VTE prophylaxis is important in preventing thrombosis of the vascular supply of a new allograft and potentially preventing graft loss. This is particularly necessary in patients with prothrombotic conditions such as a history of VTE, coagulopathy, arrhythmias, or metallic heart valves. The challenge is to use the optimum level of anticoagulation to avoid both clotting and postoperative bleeding. We report a rare case of severe postoperative retroperitoneal bleeding including psoas haematomata complicating renal transplantation.

## 2. Case Report

SM, a 55-year-old female, in established renal failure secondary to adult polycystic kidney disease, received a living unrelated renal transplant through an exchange donor program. The kidney was implanted in the left iliac fossa. Her past medical history included a mechanical aortic valve replacement, cerebrovascular accident, and ascending thoracic aortic aneurysm treated by aortic root replacement and was maintained on long-term warfarin. One week prior to her transplantation, her warfarin was replaced with enoxaparin 60 mg daily.

The donor renal vein was thin walled and cut short requiring the recipient's long saphenous vein to be harvested and used as a cuff to lengthen it. The kidney had three arteries: a small upper polar artery which was ligated, a middle artery that was anastomosed end to side to the external iliac artery, and a medium sized lower polar artery that was anastomosed end to end to the inferior epigastric artery. 

Postoperatively, she was put on a heparin infusion at a rate of 15 units/kg/hour with a targeted activated plasma thromboplastin time (APTT) ratio of 1.5–2.0. Immunosuppression comprised induction with basiliximab and maintenance on tacrolimus, mycophenolate mofetil, and prednisolone.

**Figure 1:**
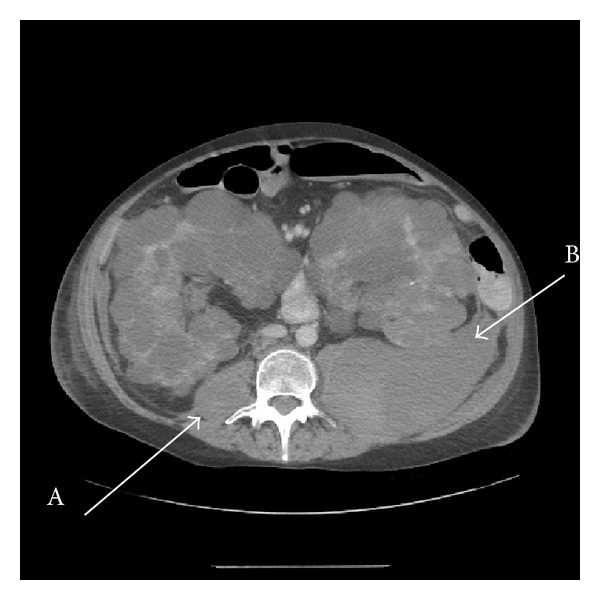
CT scan showing extensive retroperitoneal haemorrhage involving both psoas muscles (left (B) more than right (A) at this level).

A routine ultrasound scan performed on the first postoperative day showed global perfusion of the kidney, but a deep fluid collection of about 5 cm diameter was seen at the lower pole. Given an APTT ratio of 3.5, the heparin dose was reduced to 11 units/kg/hour. On the second postoperative day, she developed frank haematuria and her haemoglobin (Hb) level dropped from a preoperative level of 10.8 g/dL to 8 g/dL. A CT scan was performed which confirmed a large retroperitoneal haematoma (15 cm in diameter) with layered density but no evidence of active bleeding (Figure 1). At reexploration of the transplant, the haematoma was evacuated and the kidney was noted to be well perfused without any obvious active bleeding point. Heparin (7.5 units/kg/hour) was restarted on the next day (postoperative day three) maintaining the APTT ratio within the target range until postoperative day five when this was switched to enoxaparin 80 mg daily. The dose of enoxaparin was increased to 120 mg on day 7 as the transplant function improved.

**Figure 2:**
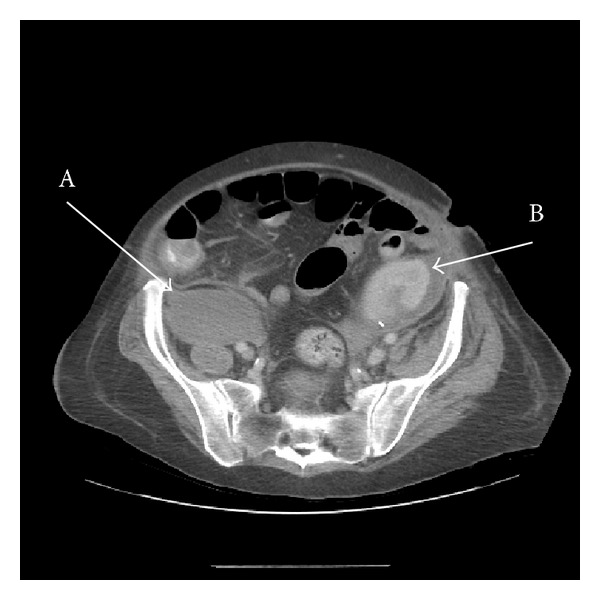
CT scan with contrast showing bilateral psoas haematomata with the right extending to a much lower level within an intact psoas muscle compartment (A: right iliopsoas haematoma; B: transplant kidney).

SM experienced several complications following transplantation including CMV viraemia, pulmonary oedema, late urine leak managed conservatively, and recurrent urinary sepsis treated with antibiotics. On postoperative day 27, she developed pedal oedema, pain in the right hip, and thigh especially on moving as well as a 3 cm swelling in the right groin. In addition, there was a significant amount of bruising in the right flank extending into the thigh and upper calf associated with paraesthesia, significant discomfort, and weakness in the right leg. She also became hypotensive (81/53 mmHg) and required resuscitation with three units of packed cells and protamine 50 mg as her Hb dropped to 4.8 g/dL and anti-Factor Xa level was 0.97. Enoxaparin was stopped. An abdominal and pelvic CT scan showed bilateral psoas haematomata (Figure 2). A repeat CT scan performed on the 31st postoperative day found no change in the haematomata apart from an extension to other retroperitoneal sites. 

By the 36th postoperative day, she was restarted on warfarin and a CT scan showed a mature haematoma (with gas locules) around the transplant kidney as well as the bilateral psoas haematomata. Linezolid was added to the antibiotic regimen and later switched to Teicoplanin based on a urine culture which grew enterococcus. Antibiotics were continued for four weeks in one form or another. Her mobility was severely impaired when she developed the psoas haematomata, effectively confining her to her bed in the initial period. Regular physiotherapy was carried out throughout and she made steady progress. On postoperative day 78, she was discharged to a local community hospital where her rehabilitation continued for a further 16 days. She then continued her convalescence at home. By eight months after her transplantation, she had returned to her pretransplant levels of fitness. At her last clinic visit (18 months after transplantation), she stated that she had returned to full fitness with regular outdoor activities and her transplant function was excellent with a creatinine of 97 *μ*mol/L (MDRD 54 mL/min).

## 3. Discussion

Iliopsoas haematoma complicating anticoagulation with warfarin or heparin is well recognised. It is usually unilateral [1], but about five cases of bilateral iliopsoas haematomata have been reported [2]. This report adds one more case of bilateral iliopsoas bleeding in a patient on long-term warfarin therapy which was converted to bridging anticoagulation with low molecular weight (LMW) heparin to allow a planned living donor renal transplantation. LMW heparins inhibit factor Xa and thrombin in the coagulation cascade and offer a more predictable anticoagulant response due to better bioavailability and dose-dependent clearance. However, it must be borne in mind that renal insufficiency delays the elimination of LMW heparins.

A meticulous regimen of anticoagulation is required particularly in the perioperative period to achieve a fine balance between preventing thrombosis and bleeding. This patient had strong indications for perioperative thromboprophylaxis due to her past history of aortic metal valve replacement, aortic root surgery, and a cerebrovascular event, which put her at increased risk of bleeding after transplantation. The challenge in her management was whether to apply heavy anticoagulation to avoid the risk and consequences of thrombosis such as loss of allograft (which had multiple vessels and a repaired vein), CVE, and cardiac complications while accepting an increased risk of bleeding. Severe bleeding could result in reoperation as in this case, requirement for transfusion (sensitisation may complicate future transplantation), wound complications, infection loss of allograft, and death. In this case, the occurrence of thromboembolism would have potentially catastrophic effects and the option to adequately prophylax against thrombosis even in the presence of bleeding was very tempting.

Bleeding into the iliopsoas apart from causing pain and bruising produces neurological problems due to its effect on the lumbar plexus, particularly the femoral nerve [2–5]. This causes difficulties of mobility and, when bilateral, could result in significant morbidity. It is not surprising that this patient required about 80 days to achieve reasonable levels of independence.

Although there was bilateral involvement of the iliopsoas muscles in this case, features of acute femoral neuropathy were predominant on the right side. This is contrary to expectation given that the kidney was implanted on the left side. Factors such as injury resulting from use of retractors during the transplant procedure and disruption of the vascular supply to the middle and distal portions of the femoral nerve which depends on the internal or external iliac artery (anastomosis of the renal transplant vessels to these may result in localised “steal”) are thought to increase the risk of acute femoral neuropathy following transplantation [6]. Haemorrhage due to LMW heparin was the dominant factor in this case and the fact that the psoas compartment was not breached on the right side might have resulted in greater compression of the femoral nerve resulting in more severe symptoms on the right side.

There is no clear consensus in the management of severe retroperitoneal haemorrhage in anticoagulated patients [4]. This case required reexploration to evacuate haematoma following severe bleeding in the early postoperative phase. However, subsequent episodes including the development of psoas haematomata were managed conservatively. The options for managing iliopsoas haematoma include conservative treatment with replacement of clotting factors, surgical declotting/decompression, and transarterial embolisation in patients with evidence of active bleeding [2, 4, 5, 7, 8].

## 4. Conclusion

Patients in established renal failure who require significant anticoagulation are at increased risk of bleedings. In the context of transplantation, this may involve prolonged hospitalisation and more protracted recovery, and patients should be carefully counselled about this.
